# Synthesis and Characterization of Cross-Linked Aggregates of Peroxidase from *Megathyrsus maximus* (Guinea Grass) and Their Application for Indigo Carmine Decolorization

**DOI:** 10.3390/molecules29112696

**Published:** 2024-06-06

**Authors:** Angie V. Perez, Jorge A. Gaitan-Oyola, Diana P. Vargas-Delgadillo, John J. Castillo, Oveimar Barbosa, Roberto Fernandez-Lafuente

**Affiliations:** 1 Grupo de Investigación en Materiales Porosos con Aplicaciones Ambientales y Tecnológicas, Departamento de Química, Universidad del Tolima, Ibagué 730006299, Colombia; avalentinaperez@ut.edu.co (A.V.P.); jarmandogaitan@ut.edu.co (J.A.G.-O.); dpvargasd@ut.edu.co (D.P.V.-D.); 2 Grupo de Investigación en Bioquímica y Microbiología, Escuela de Química, Universidad Industrial de Santander, Bucaramanga 680002, Colombia; jcasleon@uis.edu.co; 3 Departamento de Biocatálisis, ICP-CSIC, Campus Cantoblanco UAM-CSIC, C/Marie Curie 2, 28049 Madrid, Spain

**Keywords:** guinea grass peroxidase, CLEAS, indigo carmine

## Abstract

We present the synthesis of a cross-linking enzyme aggregate (CLEAS) of a peroxidase from *Megathyrsus maximus* (Guinea Grass) (GGP). The biocatalyst was produced using 50%*v*/*v* ethanol and 0.88%*w*/*v* glutaraldehyde for 1 h under stirring. The immobilization yield was 93.74% and the specific activity was 36.75 U mg^−1^. The biocatalyst surpassed by 61% the free enzyme activity at the optimal pH value (pH 6 for both preparations), becoming this increase in activity almost 10-fold at pH 9. GGP-CLEAS exhibited a higher thermal stability (2–4 folds) and was more stable towards hydrogen peroxide than the free enzyme (2–3 folds). GGP-CLEAS removes over 80% of 0.05 mM indigo carmine at pH 5, in the presence of 0.55 mM H_2_O_2_ after 60 min of reaction, a much higher value than when using the free enzyme. The operational stability showed a decrease of enzyme activity (over 60% in 4 cycles), very likely related to suicide inhibition.

## 1. Introduction

The problem of water contamination by organic pollutants such as synthetic dyes and phenolic compounds used in the pulp, paper, plastic, clothing, cosmetic, pharmaceutical, food, photography, tannery, and textile industries has become one major environmental concern, among which water pollution stands out [1,2,3,4,5,6,7]. Indigo carmine (IC) is one of the most used organic dyes, more than 33 million Kg of this compound are utilized each year [8]. Several studies have shown the harmful effects of IC, causing, for example, itching, blindness, conjunctivitis, irritation, and respiratory problems in humans [9]. It has also been shown that IC can compromise multiple organs in the human body [10]. Similarly, IC generates turbidity in water sources, blocking light, avoiding photosynthetic processes, and altering natural biological cycles [11]. IC has a complex aromatic molecular structure that gives it a high stability, making it difficult to break it down [11,12,13].

Removing organic dyes from water is essential in regulating levels of water pollutants [11,12,13]. Among the conventional techniques for the treatment of effluents contaminated with synthetic dyes, methods such as acid hydrolysis, aerobic and anaerobic biodegradation, distillation, absorption, adsorption, liquid-liquid extraction, separation by membranes (nanofiltration, reverse osmosis and pervaporation) and oxidation processes (chemical, electrochemical, photochemical and photoelectrochemical) may be highlighted [11,14,15,16]. However, these methods have a poor specificity to remove colorants, require multiple operation steps, present high costs and can generate molecules that are more toxic than the initial ones.

Peroxidases and particularly horseradish peroxidase (HRP) have gained interest due to its potential ability to degrade toxic compounds, such as IC, into compounds with a lower environmental impact [17,18,19,20,21]. However, the use of a free peroxidase to decontaminate natural water sources is complex and very expensive, with the reuse of the enzyme becoming not possible. In this context, the use of immobilized enzymes can solve some of the problems [22,23]. A heterogeneous biocatalyst can permit the use of continuous reactors compatible with enzyme reuse and the treatment of large water volumes, enabling some further enzyme features improvements [24]. Enzyme immobilization, if performed properly, may improve the stability of the enzymes via multipoint (preventing enzyme unfolding) [25] or multisubunit (preventing dissociation of multimeric enzymes) [26] covalent attachment, generation of favorable environments, and other reasons recently reviewed [27]. A proper immobilization protocol can also be coupled to enzyme purification, saving time and efforts in the preparation of an industrial biocatalyst [28]. Immobilization usually involves the alteration of the enzyme structure, and this can alter enzyme activity, selectivity, specificity, inhibitions, etc. [29]. That way, immobilization is nowadays no longer a tool directed to enzyme reuse, but another opportunity to greatly improve enzyme features for a specific process [30].

Immobilization can be performed using very different strategies, that can be divided into using pre-existing solids (that is, using a solid matrix as support) or generating an ex novo solid, without using a pre-existing support. Among these last ones, we can remark nanoflowers [31,32,33], crosslinked enzyme crystals (CLECs) [34,35,36], even though cross-linked enzyme aggregates (CLEAS) stand out as the best option [37,38,39]. This is a carrier-free enzyme immobilization methodology developed by Prof Sheldon [37,38,39]. They consist in the chemical crosslinking of enzyme aggregates, producing a solid biocatalyst that can be used under any experimental condition without risks of enzyme re-solubilization (standard enzyme aggregates can only be used in anhydrous media) [38,39]. While the precipitation of the enzyme may be generally obtained maintaining the enzyme activity by using one or another precipitant agent, it is in the chemical crosslinking where some enzymes present difficulties, mainly when they are poor in reactive groups present on their surface, making the use of some feeder (an aminated polymer, a protein with a surface rich in nucleophiles) necessary to get an efficient chemical crosslinking [40].

Peroxidase immobilization via CLEAS production has been the subject of some studies, mainly using horseradish peroxidase. These biocatalysts have been applied to the oxidation of harmful compounds [41,42,43,44]. The low HRP stability in the presence of high concentrations of H_2_O_2_ (cosubstrate of the enzyme but also an enzyme inactivating agent) [45,46,47] and other hydroperoxides has become a problem for the use of this popular enzyme [48,49]. This concern has been the main drive for the search of new sources of peroxidases, perhaps more stable versus hydrogen peroxide and thus, with better prospects of utilization. Some of them have been immobilized using CLEAs technology. For example, the peroxidase from royal palm tree (RPP) (*Roystonea regia*) has been immobilized using the CLEAS technique [50] and a magnetic-CLEA was also prepared from peroxidase extracted from cabbage leaves and this biocatalyst was able to remove high concentrations of different dyes [51].

In this new study, the preparation of a CLEA biocatalyst based on peroxidase from *Megathyrsus maximus* previously classified as *Panicum maximum* (common name Guinea Grass; the peroxidase will be called GGP) has been studied. GGP has been previously semi-purified and partially characterized [52]. Remarkably, GGP is described to be less pH- and temperature-sensitive than other peroxidases [52]. The biocatalyst was characterized under different pH, temperature and presence of organic solvents and hydrogen peroxide. The GGP-CLEAS performance in IC degradation has also been studied.

## 2. Results

### 2.1. Guinea Grass Peroxidase Cross-Linked Enzyme Aggregates (GGP-CLEAS)

The biocatalyst exhibited a specific activity of 36.75 ± 2.35 U mg^−1^ and an immobilization yield of 93.74 ± 0.05%. The specific activity of the GGP-CLEAS was 61% higher than the free enzyme (22.8 ± 1.93 U mg^−1^), suggesting an enzyme hyper-activation upon immobilization.

**Figure 1:**
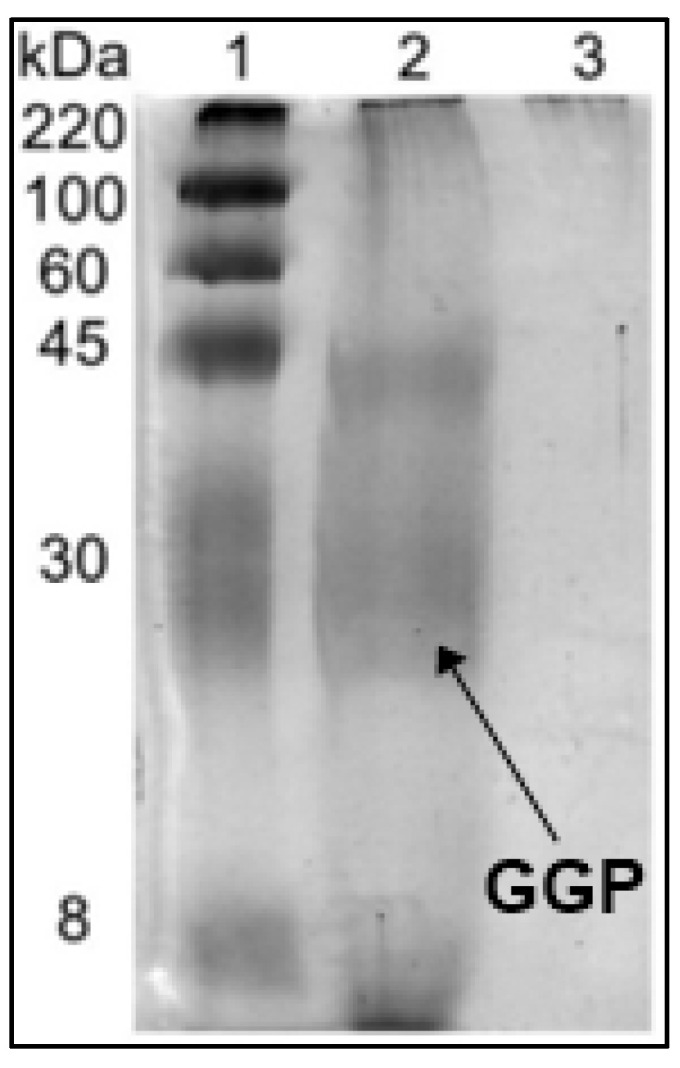
SDS-PAGE of soluble GGP and GGP-CLEAS. Lane 1: molecular weight marker (220 kDa), lane 2: soluble GGP and lane 3: GGP-CLEAs. Other specifications may be found in Methods section.

To check if the chemical crosslinking with glutaraldehyde had been effective, the GGP-CLEAS were boiled in the presence of β-mercaptoethanol/SDS and the supernatant was used to carry out a SDS-PAGE experiment (Figure 1). The untreated crude enzyme extract (lane 2) shows the characteristic GGP band at 30 kDa [52]. The band at 45 kDa indicates the presence of another protein in the crude extract [53]. However, the figure shows that there is no evidence of any protein band on the gel when using the CLEA (lane 3), confirming the success of the crosslinking of the enzyme molecules [40], not only for the peroxidase but also for the contaminant protein.

### 2.2. Characterization of the GGP-CLEAS Biocatalyst

#### 2.2.1. Effect of the pH Value on the Activity/Stability of Free and GGP-CLEAS Biocatalysts

**Figure 2:**
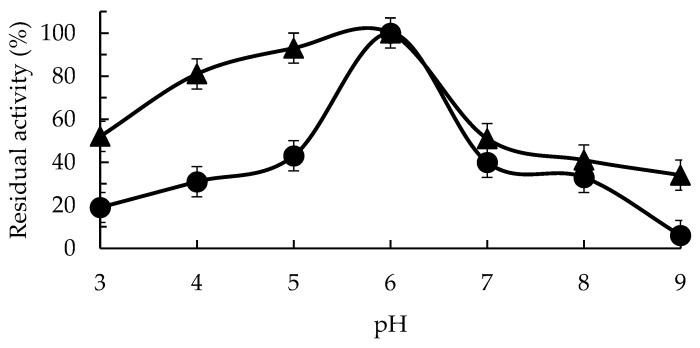
Optimum pH value for enzyme activity for GGP (circles) and GGP-CLEAS (triangles) at 25 °C. Other specifications may be found in the Methods section.

Figure 2 shows the free enzyme activity/pH profile, with a clear optimal at pH 6 and a drop in activity using lower or higher pH values (to 43% at pH 5 and 40% at pH 7). The immobilization of the enzyme via CLEAS technology permits a great increase in the activity in the acid pH region value (although the optimal pH remained 6, the activity at pH 5 was 93%). The activity of the immobilized enzyme remained over 50% even at pH 3 when the free enzyme maintained under 20% of the maximal activity at this pH value. The resistance to changes in the pH values after immobilization of the GPP activity at a pH over 6 was smaller than in the acid pH values, with just over 50% of the activity at pH 6 showing at pH 7. However, at pH 9, the immobilized enzyme retained more than 30% of the maximum activity, while the free enzyme only maintained 6% of the activity at pH 6.

Considering that the specific activity of the immobilized enzyme was higher than the free enzyme at pH 6, these results mean that the specific activity of the immobilized enzyme surpassed that of the free enzyme in all the pH ranges; this increase in activity thus becomes an advantage of the immobilized biocatalyst. This higher activity retention at extreme pH values could be caused by a higher stability of the enzyme in all the studied pH ranges. 

The free enzyme and GGP-CLEAS were incubated at 25 °C for 5 days, and the remaining activity and half-lives were determined (Table 1). The free enzyme maintained 100% of the activity at pH 3 and 4, retaining a lower percentage of initial activity when the pH increased (very slightly at pH 5–7) to reach 11% of residual activity at pH 9. That way, it was not possible to determine the half-lives of the free enzyme at pH 3 and 4, but the values were over 57 h at pH 5–7, 10.5 h at pH 8, and only 8.25 h at pH 9. The immobilized enzyme also maintained 100% of the initial activity at pH 3 and 4, showing similar decreases at pH 5–7, and the lowest activity retained was found at pH 9. The calculated half-lives were pretty similar to those of the free enzyme. At this temperature, it was not possible to detect a significant difference in the stability of the enzyme before and after immobilization. This shows that the low activity of the free enzyme, compared to the immobilized enzyme at a pH under 6, is not caused by a destabilization of the enzyme but should be related to some conformational change induced by the pH that produces a less efficient (and that way less active) but more stable enzyme form.

#### 2.2.2. Thermal Stability of GGP-CLEAs under Stress Conditions

To evaluate the thermal stability of the prepared GGP-CLEAS, different inactivation experiments were carried out at 90 °C, using the inactivation courses to calculate the half-lives of each biocatalyst (Table 2). The free enzyme exhibited the lowest stability at pH 9, and it was not possible to give any value, as the activity was 0 in the first measurement. Stability was slightly higher at pH 5 than at pH 7. Using CLEAS, again the stability at pH 9 was the lowest, and stabilities at pH 5 and 7 were the highest. The most relevant point was that in all pH values, the GPP-CLEAS biocatalyst was more stable than the free enzyme [37,39,54]. The stabilization depended on the pH value, being just over 3 at pH 5, more than 5 at pH 7, and quite higher at pH 9, suggesting that at different pH values, the inactivation causes may differ [55,56,57].

#### 2.2.3. Stability of GGP-CLEAS in the Presence of Hydrogen Peroxide

Hydrogen peroxide is one of the substrates for peroxidases. However, it is also an enzyme inactivating agent, able to produce multiple modifications of the enzyme groups, including the oxidation of the hemo group [45,46,47]. We analyzed the stability of the GGP preparations at 0.1 mM H_2_O_2_ at different pH values. The experiments were performed for 5 days (Table 3). At this time, in the presence of 0.1 mM of hydrogen peroxide, the free enzyme maintained 68% of the activity at pH 5, 19% at pH 7, and only 5% at pH 9. Immobilization improves these results. Half-lives increased by 2–3 folds upon immobilization using this hydrogen peroxide concentration.

#### 2.2.4. Stability of GGP-CLEAs in the Presence of Organic Solvents

The stability of peroxidases in organic solvents is an important factor for their use in different industrial applications [37,55]. Therefore, the stability of GGP-CLEAs was evaluated in aqueous-organic mixtures of 1,4-dioxane/100 mM Tris-HCl pH 7 or dimethyl sulfoxide/100 mM Tris-HCl pH 7.4 at a ratio 1:1 at 60 °C. The inactivation kinetics were followed, and the thermal inactivation rate constants were determined. Table 4 shows that stabilization of the enzyme caused by the immobilization was very small because the enzyme was more stable in DMSO. 

### 2.3. Use of the GGP Biocatalyst in the Decolorization of Indigo Carmine

**Figure 3:**
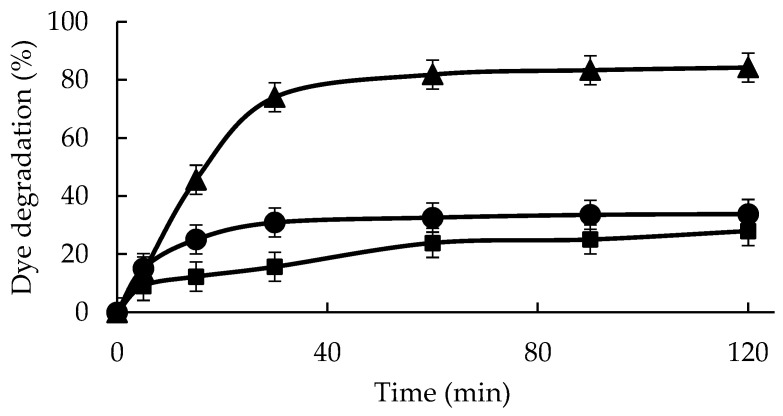
Indigo carmine (0.05 mM) degradation by GGP and GGP-CLEAS at 25 °C and pH 5 in the presence of 0.55 mM hydrogen peroxide. Reference: GGP (circles), GGP-CLEAs (triangles), and no catalyst (square). Other specifications may be found in the Methods section.

Figure 3 shows that with the direct exposure of 0.05 mM IC to 0.55 mM hydrogen peroxide at 25 °C and pH 5, the decolorization of around 28% of the IC sample was achieved after 2 h. A concentration of 0.55 mM hydrogen peroxide was used to have enough hydrogen peroxide to fully decolor the used concentration of IC, as using only 0.1 mm, the reaction stopped before reaching a significant decolorization.

The addition of a free enzyme just marginally improved this result (to 34%). The effect of the enzyme action was clearer in the first moments. The immobilized biocatalyst allowed the IC solution to decolor by 82% in 60 min, reaching 84% after 120 min at 25 °C, a much better result than using the free enzyme, although in the first moments, the free enzyme seemed to present an efficiency even higher than the immobilized enzyme. These results confirmed that the GGP biocatalysts designed in this paper greatly improve GGP performance in the reaction of IC degradation, very likely as a consequence of different factors such as an increase in enzyme stability and the generation of a more adequate conformation of the enzyme. The absorption spectra of indigo carmine, untreated and treated with GGP-CLEAS under optimal parameters, are shown in Figure S1. It is interesting that the characteristic absorption peak of the chromophore group of the dye at 610 nm disappears after 120 min of reaction catalyzed by GGP-CLEAS. Probably, the C=C bond of IC is cleaved by GGP-CLEAS, decreasing the intensity of the absorption peak and producing two molecules of 2-amino-5-(sodium benzenesulfonate)-benzoic acid. Afterwards, GGP-CLEAS release a sulfonate ion for the formation of anthranilic acid. This can be degraded into benzoic acid or aniline, which can form 4-ethyl-benzaldehyde, benzaldehyde, propanoic acid, acetic acid, or formic acid. Finally, these derivatives spontaneously degrade into water, molecular nitrogen, and carbon dioxide [58].

**Figure 4:**
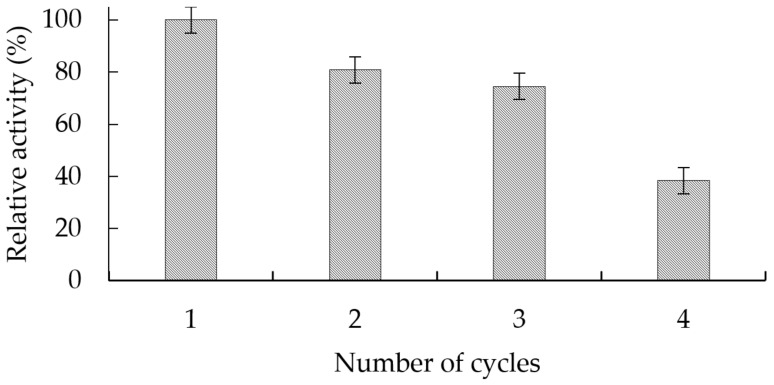
Number of reuses of GGP-CLEAS in catalytic cycles of indigo carmine degradation.

The catalytic activity of the GGP-CLEAS was evaluated in four continuous cycles of reuse in the degradation of indigo carmine, as described in Figure 3. As shown in Figure 4, GGP crosslinked aggregates after four cycles of reaction only retain 38% of the enzymatic activity exhibited in the first cycle.

## 3. Discussion

The CLEAS methodology improves the stability of the enzyme and its reusability in enzymatic cycles at the cost of a decrease in its activity [37]. The increase in specific activity compared to the free enzyme may be related to the distorting/inactivating effects of both cosubstrates in the enzyme structure that could be partially solved if the immobilization produces a more stable enzyme [27,29,59,60]. Moreover, it cannot be discarded that the use of ethanol and glutaraldehyde produced some change in the enzyme structure that led to a more active enzyme molecule structure [60]. That way, the immobilizations using this optimized CLEA strategy not only reduced the costs because they did not use any pre-existing support, but they also produced a gain in the specific activity of the enzyme.

In the catalysis of peroxidases, hydrogen peroxide can have an enzyme inactivating effect, also known as “suicide inactivation” [46,47]. This effect leads to the formation of a free radical of iron III porphyrin during the catalytic cycle of peroxidases. Therefore, two scenarios can be presented. The first corresponds to a reversible pathway, where compound III, being unstable, returns to its initial state. The second is an irreversible pathway, where compound III releases the heme group, leading to enzyme inactivation [45,55]. Hence, hydrogen peroxide is necessary for the reactions, but its excess negatively affects the stability and activity of GGP and GGP-CLEAS. Therefore, our results indicate that the formation of crosslinked enzymatic aggregates of GGP confer stability to the tertiary structure of free peroxidase, possibly preventing enzymatic inactivation by heme group loss. This result suggests that the enzyme stability must be further increased (e.g., using other immobilization techniques, using systems to partition the hydrogen peroxide from the enzyme environment) to be used under the optimal conditions. The current biocatalyst requires the use of a lower hydrogen peroxide concentration (perhaps using some feed- back configuration or a biocatalytic in situ hydrogen peroxide formation). That way, there is room for further improvements of the biocatalyst pursuing a higher operational stability of the biocatalyst.

## 4. Materials and Methods

### 4.1. Materials

Hydrogen peroxide (30%wt), (NH_4_)_2_SO_4_, 1,4-dioxane, dimethyl sulfoxide, glutaraldehyde, indigo carmine (IC), Tris base, HCl (37%wt), glycerol, bromophenol blue, and Coomassie brilliant blue R-250 were purchased from Merck. Ethanol was from Supelco. Poly(ethylenglycol) (PEG) (MW 1000), guaiacol, sodium dodecyl sulfate (SDS), and β-mercaptoethanol were from Sigma-Aldrich. All reagents and solvents were of analytical grade. 

### 4.2. Peroxidase Extraction

Leaves of Guinea Grass were harvested in Alexander Von Humboldt Botanical Garden (Ibague, Tolima, Colombia). Only green and undamaged leaves were used. The extraction of peroxidase from Guinea Grass leaves was performed as previously described with some modifications [61]. First, the harvested leaves were cut in small pieces (5 cm) and mashed in 10 mM phosphate buffered saline solution (PBS) at pH 6.0, keeping the washing solutions containing the enzyme. The obtained extracts were incubated in a two-phase system formed by 14% PEG and 10% (NH_4_)_2_SO_4_. The aqueous phase containing most of the GGP was further submitted to a gel permeation chromatography by using a Sephadex G-50 column to semi-purify the enzyme. 

### 4.3. SDS-PAGE Electrophoresis

The soluble enzyme and GGP-CLEAS were analyzed using the sodium dodecyl sulfate polyacrylamide gel electrophoresis (SDS-PAGE) technique. A total of 50 µL GGP or 20 mg GGP-CLEAS was incubated with lysis buffer at 100 °C for 5 min, centrifuged for 10 s at 12,000 rpm, and cooled on an ice bed for 5 min. Subsequently, 10 µL molecular weight marker (80–220 kDa), GGP, and GGP-CLEAS were deposited in 12% polyacrylamide gel, and the proteins were separated at a constant voltage of 100 V at 25 °C for 1 h. Protein bands were visualized by subjecting the gel to Coomassie brilliant blue R-250 staining for 1 h. The lysis buffer contained 0.5 mM Tris-HCL pH 6.8, 2.5% sodium dodecyl sulfate (SDS), 25% β-mercaptoethanol, 25% glycerol, and 0.1 mg mL^−1^ bromophenol blue.

### 4.4. Determination of Enzyme Activity

Standard enzyme activity determination was measured adding 100 µL of the enzyme solution or 100 mg GGP-CLEAS to 1.96 mL of 10 mM PBS at pH 6.0 containing 18.2 mM guaiacol and 4.4 mM H_2_O_2_ as substrates, measuring the increase in the absorbance at 470 nm promoted by the formation of tetraguaiacol (ε = 5200 M^−1^ cm^−1^) [61]. One international unit of activity (U) was defined as the amount of peroxidase that oxidases 1 µmol of guaiacol per minute. Protein concentration was quantified by Bradford’s method [62], employing bovine serum albumin as a reference. 

### 4.5. Cross-Linked Enzyme Aggregate Preparation

A volume of 3 mL of 50%*v*/*v* ethanol was added to 1 mL of GGP solution (0.62 mg mL^−1^) at 4 °C under constant stirring (300 rpm). After 1 h, glutaraldehyde 0.88%*w*/*v* was added, and the reaction mixture was incubated for 1 h. GGP-CLEAS were recovered by centrifugation, washed 3 times with 10 volumes of 10 mM sodium phosphate buffer at pH 6, and stored at 4 °C. 

### 4.6. Optimum pH Value Determination of GGP and GGP-CLEAS

Determination of the optimal pH for GGP and GGP-CLEAS enzyme activity was evaluated. A 100 µL GGP solution or 100 mg GGP-CLEAS was suspended in 1.96 mL of 100 mM citric acid buffer at pH 3, 100 mM sodium acetate buffer at pH 4–5, 100 mM sodium citrate buffer at pH 6, 100 mM sodium phosphate buffer at pH 7–8, or 100 mM sodium carbonate buffer at pH 9, containing 18.2 mM guaiacol and 4.4 mM H_2_O_2_ as substrates at 25 °C, immediately measuring the absorbance using guaiacol as a substrate, as described above.

### 4.7. Effect of pH on the Stability of Different GGP Preparations 

The effect of pH on the GGP and GGP-CLEAS activity was evaluated. A 100 µL GGP solution or 100 mg GGP-CLEAS was suspended in 5 mL of 100 mM citric acid buffer at pH 3, 100 mM sodium acetate buffer at pH 4–5, 100 mM sodium citrate buffer at pH 6, 100 mM sodium phosphate buffer at pH 7–8, or 100 mM sodium carbonate buffer at pH 9, under constant stirring (30 rpm) for 5 days at 25 °C. Samples of these suspensions were taken periodically, and the enzymatic activity was measured using guaiacol as a substrate, as described above. Half-lives were calculated from the observed inactivation courses.

### 4.8. Stress Thermal Inactivation of Different GGP Preparations

For thermal inactivation experiments, a 100 µL GGP solution or 100 mg GGP-CLEAS was suspended in 100 mM sodium acetate buffer at pH 5, 100 mM sodium phosphate buffer at pH 7, or 100 mM sodium carbonate buffer at pH 9 at 90 °C under constant stirring (30 rpm). Samples of these inactivating suspensions were taken periodically, and the enzymatic activity was measured using guaiacol as a substrate, as described above. Half-lives were calculated from the observed inactivation courses.

### 4.9. Inactivation in the Presence of Hydrogen Peroxide of Different GGP Preparations

The inactivation effect of H_2_O_2_ was determined by adding a 100 µL GGP solution or 100 mg GGP-CLEAS to 5 mL of 100 mM sodium acetate buffer at pH 5, 100 mM sodium phosphate buffer at pH 7, or 100 mM sodium carbonate buffer at pH 9 at 0.1 mM H_2_O_2_ with 25 °C under constant stirring (30 rpm) for 5 days. Samples were taken periodically, and the enzymatic activity was measured using guaiacol as a substrate, as described above. Half-lives were calculated from the observed inactivation courses.

### 4.10. Inactivation in the Presence of Organic Solvents of Different GGP Preparations

The inactivation courses of GGP and GGP-CLEAS in the presence of organic solvents were determined by adding a 100 µL GGP solution or 100 mg GGP-CLEAS to 5 mL of 1,4-dioxane/100 mM Tris-HCl pH 7 or dimethyl sulfoxide/100 mM Tris-HCl pH 7.4 at a ratio 1:1 at 60 °C under constant stirring (30 rpm). 

### 4.11. Indigo Carmine Degradation Using GGP-CLEAS

A volume of 2 mL of 0.05 mM IC solution at pH 5 was added to a 100 µL GGP solution or 100 mg GGP-CLEAS with 0.55 mM H_2_O_2_ at 25 °C under dark conditions and under constant stirring (30 rpm) for 2 h. IC solutions incubated with hydrogen peroxide in the absence of a biocatalyst at identical concentrations were used as a blank [50,58]. Dye degradation was monitored spectrophotometrically at 610 nm.

### 4.12. GGP-CLEAS Operational Stability

Reusability of optimal GGP-CLEAS on IC degradation was evaluated. A total of 100 mg of GGP-CLEAS was suspended in a solution of 10 mL of 0.05 mM IC at pH 5 containing 0.55 mM hydrogen peroxide at 25 °C in the dark and under constant stirring (30 rpm) for 2 h. Once the reaction was completed, the GGP-CLEAS were taken from the reaction mixture, washed with 10 mM sodium phosphate at pH 6, and used again in a fresh IC solution. Dye degradation was monitored spectrophotometrically at 610 nm.

## 5. Conclusions

This work details the preparation of CLEAS of the novel peroxidase from *Megathyrsus maximus* and its application for indigo carmine degradation. The GGP-CLEAS exhibited an increase in their specific activity by a factor of 1.6 times compared to native GGP at pH 6, but this gain may be multiplied even by six under certain conditions (e.g., pH 9). The GGP-CLEAS showed a higher catalytic efficiency and stability compared to the soluble peroxidase. Therefore, the GGP-CLEAS showed a high efficiency in the decolorization of IC, reaching 84% of degradation. The moderate operational stability of the biocatalysts under optimized conditions still requires some attention; other immobilization strategies or modifications of the enzyme may permit further improvements of the enzyme operational stability. Moreover, other reactor configurations may solve, at least partially, the current problems.

## Figures and Tables

**Table 1:** Half-life of GGP and optimal GGP-CLEAS at 25 °C at different pH values after 5 days.

pH	t ½ (min)	
	GGP	GGP-CLEAS
3	No activity loss after 5 days	No activity loss after 5 days
4	No activity loss after 5 days	No activity loss after 5 days
5	3466 ± 87	6931 ± 134
6	3466 ± 98	3466 ± 84
7	3466 ± 65	3466 ± 129
8	630.1 ± 17.1	770.2 ± 71.2
9	495.1 ± 9.7	693.2 ± 21.2

**Table 2:** Half-life of GGP and optimal GGP-CLEAS at 90 °C.

pH	t ½ (min)	
	GGP	GGP-CLEAS
5	0.922 ± 0.023	2.95 ± 0.32
7	0.785 ± 0.012	4.09 ± 0.23
9	Activity was 0 in the first measure	1.78 ± 0.12

**Table 3:** Half-life times according to hydrogen peroxide concentration for GGP and GGP-CLEAS at 25 °C in the presence of 0.1 mM hydrogen peroxide.

pH	t ½ (min)	
	GGP	GGP-CLEAS
5	9902	17,329
7	2310	6931
9	1155	3466

**Table 4:** Inactivation constant by organic solvents for GGP and GGP-CLEAS at 60 °C.

pH	k_i_ (min^−1^)	
	GGP	GGP-CLEAS
1,4-dioxane	7.72 ± 0.41 × 10^−1^	5.05 ± 0.43 × 10^−1^
DMSO	4.88 ± 0.32 × 10^−2^	4.68 ± 0.24 × 10^−2^

## Data Availability

The data used in this paper may be obtained from the Authors.

## Supplementary Materials

The following supporting information can be downloaded at: https://www.mdpi.com/article/10.3390/molecules29112696/s1, Figure S1: Indigo carmine UV spectrum before and after GGP-CLEAS treatment.

## References

[1] Bilal M., Iqbal H.M. (2019). Lignin peroxidase immobilization on Ca-alginate beads and its dye degradation performance in a packed bed reactor system. Biocatal. Agric. Biotechnol..

[2] Doherty A.-C., Lee C.-S., Meng Q., Sakano Y., Noble A.E., Grant K.A., Esposito A., Gobler C.J., Venkatesan A.K. (2023). Contribution of household and personal care products to 1,4-dioxane contamination of drinking water. Curr. Opin. Environ. Sci. Health.

[3] Wang R., Cheng H., Gong Y., Huang T. (2023). New brominated flame retardant decabromodiphenyl ethane (DBDPE) in water sediments: A review of contamination characteristics, exposure pathways, ecotoxicological effects and health risks. Environ. Pollut..

[4] Kodešová R., Švecová H., Klement A., Fér M., Nikodem A., Fedorova G., Rieznyk O., Kočárek M., Sadchenko A., Chroňáková A. (2024). Contamination of water, soil, and plants by micropollutants from reclaimed wastewater and sludge from a wastewater treatment plant. Sci. Total Environ..

[5] Fehrenbach G.W., Pogue R., Carter F., Clifford E., Rowan N. (2022). Implications for the seafood industry, consumers and the environment arising from contamination of shellfish with pharmaceuticals, plastics and potentially toxic elements: A case study from Irish waters with a global orientation. Sci. Total Environ..

[6] He Y., Zhang Y., Ju F. (2022). Metformin Contamination in Global Waters: Biotic and Abiotic Transformation, Byproduct Generation and Toxicity, and Evaluation as a Pharmaceutical Indicator. Environ. Sci. Technol..

[7] Srivastav A.L., Patel N., Rani L., Kumar P., Dutt I., Maddodi B.S., Chaudhary V.K. (2023). Sustainable options for fertilizer management in agriculture to prevent water contamination: A review. Environ. Dev. Sustain..

[8] Qu R., Xu B., Meng L., Wang L., Wang Z. (2015). Ozonation of indigo enhanced by carboxylated carbon nanotubes: Performance optimization, degradation products, reaction mechanism and toxicity evaluation. Water Res..

[9] Ahmad M.B., Soomro U., Muqeet M., Ahmed Z. (2020). Adsorption of Indigo Carmine dye onto the surface-modified adsorbent prepared from municipal waste and simulation using deep neural network. J. Hazard. Mater..

[10] Neves M.I.L., Silva E.K., Meireles M.A.A. (2021). Natural blue food colorants: Consumer acceptance, current alternatives, trends, challenges, and future strategies. Trends Food Sci. Technol..

[11] Chowdhury M.F., Khandaker S., Sarker F., Islam A., Rahman M.T., Awual M.R. (2020). Current treatment technologies and mechanisms for removal of indigo carmine dyes from wastewater: A review. J. Mol. Liq..

[12] Reyes-Márquez V., Rojas L.E.C., Colorado-Peralta R., Peña-Rodríguez R., Rivera-Villanueva J.M., Morales-Morales D. (2023). Adsorption potential of polymeric porous crystalline materials (MOFs) for the removal of Indigo carmine, Congo red, and Malachite green from water. Inorganica Chim. Acta.

[13] Ristea M.-E., Zarnescu O. (2023). Indigo Carmine: Between Necessity and Concern. J. Xenobiotics.

[14] Katheresan V., Kansedo J., Lau S.Y. (2018). Efficiency of various recent wastewater dye removal methods: A review. J. Environ. Chem. Eng..

[15] Ben Slama H., Bouket A.C., Pourhassan Z., Alenezi F.N., Silini A., Cherif-Silini H., Oszako T., Luptakova L., Golińska P., Belbahri L. (2021). Diversity of Synthetic Dyes from Textile Industries, Discharge Impacts and Treatment Methods. Appl. Sci..

[16] Alshabib M., Onaizi S.A. (2019). A review on phenolic wastewater remediation using homogeneous and heterogeneous enzymatic processes: Current status and potential challenges. Sep. Purif. Technol..

[17] Silva D., Rodrigues C.F., Lorena C., Borges P.T., Martins L.O. (2023). Biocatalysis for biorefineries: The case of dye-decolorizing peroxidases. Biotechnol. Adv..

[18] Sellami K., Couvert A., Nasrallah N., Maachi R., Abouseoud M., Amrane A. (2021). Peroxidase enzymes as green catalysts for bioremediation and biotechnological applications: A review. Sci. Total Environ..

[19] Basumatary D., Yadav H.S., Yadav M. (2023). The Role of Peroxidases in the Bioremediation of Organic Pollutants. Nat. Prod. J..

[20] Saikia S., Yadav M., Hoque R.A., Yadav H.S. (2023). Bioremediation mediated by manganese peroxidase—An overview. Biocatal. Biotransform..

[21] Khalid N., Kalsoom U., Ahsan Z., Bilal M. (2022). Non-magnetic and magnetically responsive support materials immobilized peroxidases for biocatalytic degradation of emerging dye pollutants—A review. Int. J. Biol. Macromol..

[22] DiCosimo R., McAuliffe J., Poulose A.J., Bohlmann G. (2013). Industrial use of immobilized enzymes. Chem. Soc. Rev..

[23] Liese A., Hilterhaus L. (2013). Evaluation of immobilized enzymes for industrial applications. Chem. Soc. Rev..

[24] Mateo C., Palomo J.M., Fernandez-Lorente F., Guisan J.M., Fernandez-Lafuente R. (2007). Improvement of enzyme activity, stability and selectivity via immobilization techniques. Enzym. Microb. Technol..

[25] Klibanov A.M. (1983). Stabilization of Enzymes against Thermal Inactivation. Adv. Appl. Microbiol..

[26] Fernandez-Lafuente R. (2009). Stabilization of multimeric enzymes: Strategies to prevent subunit dissociation. Enzym. Microb. Technol..

[27] Rodrigues R.C., Berenguer-Murcia Á., Carballares D., Morellon-Sterling R., Fernandez-Lafuente R. (2021). Stabilization of enzymes via immobilization: Multipoint covalent attachment and other stabilization strategies. Biotechnol. Adv..

[28] Barbosa O., Ortiz C., Berenguer-Murcia Á., Torres R., Rodrigues R.C., Fernandez-Lafuente R. (2015). Strategies for the one-step immobilization–purification of enzymes as industrial biocatalysts. Biotechnol. Adv..

[29] Rodrigues R.C., Ortiz C., Berenguer-Murcia Á., Torres R., Fernández-Lafuente R. (2013). Modifying enzyme activity and selectivity by immobilization. Chem. Soc. Rev..

[30] Bolivar J.M., Woodley J.M., Fernandez-Lafuente R. (2022). Is enzyme immobilization a mature discipline? Some critical considerations to capitalize on the benefits of immobilization. Chem. Soc. Rev..

[31] Chen J., Guo Z., Xin Y., Gu Z., Zhang L., Guo X. (2023). Organic–Inorganic Hybrid Nanoflowers: A Comprehensive Review of Current Trends, Advances, and Future Perspectives. Coord. Chem. Rev..

[32] Jafari-Nodoushan H., Mojtabavi S., Faramarzi M.A., Samadi N. (2022). Organic-inorganic hybrid nanoflowers: The known, the unknown, and the future. Adv. Colloid Interface Sci..

[33] da Costa F.P., Cipolatti E.P., Junior A.F., Henriques R.O. (2022). Nanoflowers: A New Approach of Enzyme Immobilization. Chem. Rec..

[34] Kojima M., Abe S., Ueno T. (2021). Engineering of protein crystals for use as solid biomaterials. Biomater. Sci..

[35] Staar M., Schallmey A. (2023). Performance of cross-linked enzyme crystals of engineered halohydrin dehalogenase HheG in different chemical reactor systems. Biotechnol. Bioeng..

[36] Staar M., Staar S., Schallmey A. (2022). Crystal Contact Engineering for Enhanced Cross-Linking Efficiency of HheG Crystals. Catalysts.

[37] Sheldon R.A. (2019). CLEAs, Combi-CLEAs and ‘Smart’ Magnetic CLEAs: Biocatalysis in a Bio-Based Economy. Catalysts.

[38] Cao L., van Rantwijk F., Sheldon R.A. (2000). Cross-Linked Enzyme Aggregates: A Simple and Effective Method for the Immobilization of Penicillin Acylase. Org. Lett..

[39] Schoevaart R., Wolbers M., Golubovic M., Ottens M., Kieboom A., van Rantwijk F., van der Wielen L., Sheldon R. (2004). Preparation, optimization, and structures of cross-linked enzyme aggregates (CLEAs). Biotechnol. Bioeng..

[40] Sampaio C.S., Angelotti J.A., Fernandez-Lafuente R., Hirata D.B. (2022). Lipase immobilization via cross-linked enzyme aggregates: Problems and prospects—A review. Int. J. Biol. Macromol..

[41] Ayhan F., Akpolat O. (2021). Experimental Design Optimization and Decolorization of an Azo Dye by Cross-Linked Peroxidase Aggregates. Tekst- VE Konfeksiyon.

[42] Bilal M., Iqbal H.M., Hu H., Wang W., Zhang X. (2017). Development of horseradish peroxidase-based cross-linked enzyme aggregates and their environmental exploitation for bioremediation purposes. J. Environ. Manag..

[43] Šekuljica N.Ž., Prlainović N.Ž., Jakovetić S.M., Grbavčić S.Ž., Ognjanović N.D., Knežević-Jugović Z.D., Mijin D.Ž. (2016). Removal of Anthraquinone Dye by Cross-Linked Enzyme Aggregates From Fresh Horseradish Extract. Clean-Soil Air Water.

[44] Šulek F., Fernández D.P., Knez Ž, Habulin M., Sheldon R.A. (2011). Immobilization of horseradish peroxidase as crosslinked enzyme aggregates (CLEAs). Process. Biochem..

[45] de Oliveira F.K., Santos L.O., Buffon J.G. (2021). Mechanism of action, sources, and application of peroxidases. Food Res. Int..

[46] Basha S.A., Rao U.J.P. (2017). Purification and characterization of peroxidase from sprouted green gram (*Vigna radiata*) roots and removal of phenol and *p*-chlorophenol by immobilized peroxidase. J. Sci. Food Agric..

[47] Svetozarević M., Šekuljica N., Knežević-Jugović Z., Mijin D. (2020). Agricultural waste as a source of peroxidase for wastewater treatment: Insight in kinetics and process parameters optimization for anthraquinone dye removal. Environ. Technol. Innov..

[48] Hamid M., Khalil-ur-Rehman (2009). Potential applications of peroxidases. Food Chem..

[49] Ryan B.J., Ó’Fágáin C. (2007). Effects of single mutations on the stability of horseradish peroxidase to hydrogen peroxide. Biochimie.

[50] Morales A., Barbosa O., Rueda N., Fonseca Z., Torres R., Rodrigues R.C., Ortiz C., Fernandez-Lafuente R. (2015). Optimization and characterization of CLEAs of the very thermostable dimeric peroxidase from Roystonea regia. RSC Adv..

[51] Mehde A.A. (2019). Development of magnetic cross-linked peroxidase aggregates on starch as enhancement template and their application for decolorization. Int. J. Biol. Macromol..

[52] Centeno D.A., Solano X.H., Castillo J.J. (2017). A new peroxidase from leaves of guinea grass (*Panicum maximum*): A potential biocatalyst to build amperometric biosensors. Bioelectrochemistry.

[53] Feltrin A.C.P., Fontes M.R.V., Gracia H.D.K., Badiale-Furlong E., Garda-Buffon J. (2017). Peroxidase from soybean meal: Obtention, purification and application in reduction of deoxynivalenol levels. Química Nova.

[54] Sheldon R.A., Basso A., Brady D. (2021). New frontiers in enzyme immobilisation: Robust biocatalysts for a circular bio-based economy. Chem. Soc. Rev..

[55] Oztekin A., Tasbasi S. (2020). A novel peroxidase from runner bean (*Phaseolus coccineus* L.): Enhanced affinity purification, characterization, and dye decolorization activity. J. Food Biochem..

[56] Sanchez A., Cruz J., Rueda N., dos Santos J.C.S., Torres R., Ortiz C., Villalonga R., Fernandez-Lafuente R. (2016). Inactivation of immobilized trypsin under dissimilar conditions produces trypsin molecules with different structures. RSC Adv..

[57] Souza P.M.P., Carballares D., Gonçalves L.R.B., Fernandez-Lafuente R., Rodrigues S. (2022). Immobilization of Lipase B from *Candida antarctica* in Octyl-Vinyl Sulfone Agarose: Effect of the Enzyme-Support Interactions on Enzyme Activity, Specificity, Structure and Inactivation Pathway. Int. J. Mol. Sci..

[58] Terres J., Battisti R., Andreaus J., de Jesus P.C. (2014). Decolorization and degradation of Indigo Carmine dye from aqueous solution catalyzed by horseradish peroxidase. Biocatal. Biotransform..

[59] Fernandez–Lafuente R., Guisan J.M., Ali S., Cowan D. (2000). Immobilization of functionally unstable catechol-2,3-dioxygenase greatly improves operational stability. Enzym. Microb. Technol..

[60] Boudrant J., Woodley J.M., Fernandez-Lafuente R. (2020). Parameters necessary to define an immobilized enzyme preparation. Process. Biochem..

[61] Sakharov I., Vesgac B.M.V., Galaev I., Sakharova I., Pletjushkina O. (2001). Peroxidase from leaves of royal palm tree Roystonea regia: Purification and some properties. Plant Sci..

[62] Bradford M. (1976). A Rapid and Sensitive Method for the Quantitation of Microgram Quantities of Protein Utilizing the Principle of Protein-Dye Binding. Anal. Biochem..

