# Supplementary material for: Synthesis and Characterization of Cross-Linked Aggregates of Peroxidase from Megathyrsus maximus (Guinea Grass) and Their Application for Indigo Carmine Decolorization
Source: Molecules. 2024 Jun 6;29(11):2696. doi: 10.3390/molecules29112696 (PMC11173754; doi:10.3390/molecules29112696)
Supplement: Supplementary file 1 [file molecules-29-02696-s001.zip › molecules-2990065-supplementary.pdf]

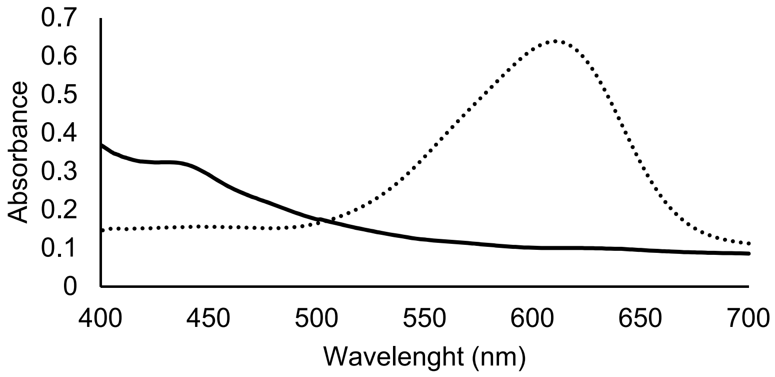

Figure S1. Indigo carmine UV spectrum before and after GGP-CLEAS treatment. Reference No treatment (dashed lines) and GGP-CLEAs treatment (solid lines).
